# Supplementary material for: Antagonistic regulation by insulin-like peptide and activin ensures the elaboration of appropriate dendritic field sizes of amacrine neurons
Source: eLife. 2020 Mar 16;9:e50568. doi: 10.7554/eLife.50568 (PMC7075694; doi:10.7554/eLife.50568)
Supplement: Source code 1. [file elife-50568-code1.zip › Readme-programs for Figure 7.docx]

neuronclass.py:

a program that defines “neurons” as a class, and the associated elements for statistics, used by stochastic.py and scan.py

scan.py

a program that generates the data in Figure 7A-C

heatmap_collect.py

makes the plots (Figure 7A-C).

stochastic.py

a program that generates data in Figure 7D. Parameter values are also included in the program.

boxplot.py

makes the plot (Figure 7D).

Model parameters:

For Figure 7D they are:

|  | Branching freq. | Terminating Freq. | Switch length |
| --- | --- | --- | --- |
| Tm20 | 0.360 | 0.594 |  |
| Babo Tm20 | 0.322 | 0.431 |  |
| Tm2 | 0.393 | 0.602 |  |
| Tm9 | 0.371 | 0.589 |  |
| Δk=0.001 | 0.360 | 0.361 |  |
| Δk=0.002 | 0.360 | 0.362 |  |
| Δk=0.005 | 0.360 | 0.365 |  |
| Two-stage | 0.42, 0,36 | 0.40, 0.40 | 100 |
| Two-stage 2 | 0.42, 0.36 | 0.40, 0.42 | 100 |

For figure 7A-C, the parameters were as scanned, each with 100 samples.
