# Supplementary material for: Antagonistic regulation by insulin-like peptide and activin ensures the elaboration of appropriate dendritic field sizes of amacrine neurons
Source: eLife. 2020 Mar 16;9:e50568. doi: 10.7554/eLife.50568 (PMC7075694; doi:10.7554/eLife.50568)
Supplement: Supplementary file 1. [file elife-50568-supp1.docx]

| Figures | Genotype |
| --- | --- |
| 1C-C’ | *hsFLP^122^/yw; FRT40/ FRT40, Tub-GAL80; 24F06-GAL4, UAS-mCD8GFP/+* |
| 1D-D’ | *hsFLP^122^/yw; FRT40, Tor*^Δ^*^P^/ FRT40, Tub-GAL80; 24F06-GAL4, UAS-mCD8GFP/+* |
| 1E-E’ | *hsFLP^122^/yw; FRT40, Tor*^Δ^*^P^/ FRT40, Tub-GAL80; DIP-*γ*-GAL4, UAS-mCD8GFP/UAS-Tor^WT^* |
| 1F-F’ | *hsFLP^122^/yw; Ort^C2b^-GAL4, UAS-mCD8GFP/+; FRT82, Rheb^3M2^/ FRT82, Tub-GAL80* |
| 1G-G’ | *hsFLP^122^/yw; Ort^C2b^-GAL4, UAS-mCD8GFP/+; FRT82, Tsc1^1A2^/ FRT82, Tub-GAL80* |
| 2A | *hsFLP^122^/yw; FRT40/ FRT40, Tub-GAL80; 24F06-GAL4, UAS-mCD8GFP/+* |
| 2B | *hsFLP^122^/yw; Ort^C2b^-GAL4, UAS-mCD8GFP/+; FRT82, InR^273^/ FRT82, Tub-GAL80* |
| 2C | *hsFLP^122^/yw; FRT40, chico^Fs(2)4^/ FRT40,Tub-GAL80; 24F06-GAL4, UAS-mCD8GFP/+* |
| 2D | *hsFLP^122^/yw; Ort^C2b^-GAL4, UAS-mCD8GFP/+; FRT82, Df(3R)PI3K92E^A^/ FRT82,Tub-GAL80* |
| 2E | *hsFLP^122^/yw; FRT40, Pten^2L117^/ FRT40, Tub-GAL80; 24F06-GAL4, UAS-mCD8GFP/+* |
| 2F | *hsFLP^122^/yw; Ort^C2b^-GAL4, UAS-mCD8-GFP/ +; FRT2A, SREBP^189^/FRT2A, Tub-GAL80* |
| 2G | *hsFLP^122^/yw; FRT40 / FRT40, Tub-GAL80; DIP-*γ*-GAL4, UAS-mCD8GFP/UAS-SREBP^WT^* |
| 2H | *hsFLP^122^/UAS-SREBP^CA^; FRT40 / FRT40, Tub-GAL80; DIP-*γ*-GAL4, UAS-mCD8GFP/+* |
| 2I | *hsFLP^122^/yw; FRT40, Tor*^Δ^*^P^/ FRT40, Tub-GAL80; DIP-*γ*-GAL4, UAS-mCD8GFP/UAS-SREBP^WT^* |
| 2J | *hsFLP^122^/UAS-SREBP^CA^; FRT40, Tor*^Δ^*^P^/ FRT40, Tub-GAL80; DIP-*γ*-GAL4, UAS-mCD8GFP/+* |
| 2K | *hsFLP1, Tub-Gal80, FRT19A/Raptor^Del^; FRT19A; +/+; Ort^C2b^-GAL4, UAS- myr-TdTomato/+* |
| 2L | *hsFLP^122^; FRT40, Tor*^Δ^*^P^/ FRT40, Tub-GAL80; DIP-*γ*-GAL4, UAS-mCD8GFP/UAS-InR^K1409A^* |
| 2M | *hsFLP^122^; FRT40 / FRT40, Tub-GAL80; DIP-*γ*-GAL4, UAS-mCD8GFP/UAS-InR^K1409A^* |
| 3B | *hsFLP^122^/yw; FRT40/ FRT40, Tub-GAL80; 24F06-GAL4, UAS-mCD8Cherry /GMR-CD4-spGFP1-10, UAS-Ort-HA-spGFP11* |
| 3C | *hsFLP^122^/yw; FRT40, Tor*^Δ^*^P^/ FRT40, Tub-GAL80; 24F06-GAL4, UAS-mCD8Cherry / GMR-CD4-spGFP1-10, UAS-Ort-HA-spGFP11* |
| 3D | *hsFLP^122^/yw; FRT40, Pten^2L117^/ FRT40, Tub-GAL80; 24F06-GAL4, UAS-mCD8Cherry /GMR-CD4-spGFP1-10, UAS-Ort-HA-spGFP11* |
| 3E | *hsFLP^122^/yw; FRT40, chico^fs(2)4^/ FRT40,Tub-GAL80; 24F06-GAL4, UAS-mCD8Cherry /GMR-CD4-spGFP1-10, UAS-Ort-HA-spGFP11* |
| 3G&L&Q | *hsFLP^122^/yw; FRT40/ FRT40, Tub-GAL80; 24F06-GAL4, UAS-mCD8Cherry /Rh3/4-Syb-spGFP1-10, UAS-Ort-HA-spGFP11* |
| 3H&M | *hsFLP^122^/yw; FRT40, Tor*^Δ^*^P^/ FRT40, Tub-GAL80; 24F06-GAL4, UAS-mCD8Cherry /Rh3/4-Syb-spGFP1-10, UAS-Ort-HA-spGFP11* |
| 3I&N | *hsFLP^122^/yw; FRT40, Pten^2L117^/ FRT40, Tub-GAL80; 24F06-GAL4, UAS-mCD8Cherry /Rh3/4-Syb-spGFP1-10, UAS-Ort-HA-spGFP11* |
| 3J&O | *hsFLP^122^/yw; FRT40, chico^fs(2)4^/ FRT40,Tub-GAL80; 24F06-GAL4, UAS-mCD8Cherry /Rh3/4-Syb-spGFP1-10, UAS-Ort-HA-spGFP11* |
| 3R-T | *hsFLP^122^/yw; FRT40, Pten^2L117^/ FRT40, Tub-GAL80; 24F06-GAL4, UAS-mCD8Cherry /Rh3/4-Syb-spGFP1-10, UAS-Ort-HA-spGFP11* |
| 4D-D’ | *GMR-GAL4, UAS-RFP; +/+; InR-V5-spGFP11/+* |
| 4E-J’ | *hsFLP^1^/yw; UAS-FSF-myr-TdTomato-T2A-spGFP1-10/+; DIP-*γ*-GAL4/InR-V5-spGFP11* |
| 5B-B’’’ | *yw; UAS-Dilp2-GFP/+; GMR9B08-GAL4/+* |
| 5C-C’ | *yw; UAS-Dilp2-GFP/+; GMR9B08-GAL4, UAS-myr-tdTom/+* |
| 5D-E’ | *yw: +; +* |
| 5F-G’ | *yw; UAS-mCD8GFP /+; L5^6-60^-GAL4/+* |
| 5H | *hsFLP^1^/UAS-Dicer2; 24F06-LexA/+; LexAop2-FSF-mCD8GFP/Tm2* |
| 5I | *hsFLP^1^/ UAS-Dicer2; UAS-Dilp2-RNAi/24F06-LexA; LexAop2-FSF-mCD8GFP/GMR9B08-GAL4* |
| 5J | *hsFLP^1^/ UAS-Dicer2; UAS-Dilp2-RNAi/24F06-LexA; LexAop2-FSF-mCD8GFP/GMR27G05-GAL4* |
| 5K | *hsFLP^1^/UAS-Dicer2; UAS-Dilp2-RNAi/24F06-LexA; LexAop2-FSF-mCD8GFP/L5^6-60^-GAL4* |
| 5L | *hsFLP^1^/ UAS-Dicer2; UAS-Dilp2-RNAi/24F06-LexA; LexAop2-FSF-mCD8GFP/GMR-GAL4* |
| 5M | *hsFLP^1^/ UAS-Dicer2; UAS-Dilp6-Ri/24F06-LexA; LexAop2-FSF-mCD8GFP/GMR-GAL4* |
| 5N | *hsFLP^1^/yw; UAS-Dilp2/24F06-LexA; LexAop2-FSF-mCD8GFP/L5^6-60^-GAL4* |
| 5O | *hsFLP^1^/yw; UAS-Dilp2/24F06-LexA; LexAop2-FSF-mCD8GFP/GMR-GAL4* |
| 6B | *hsFLP^122^, sev^E2^; FRT40, Tor*^Δ^*^P^/ FRT40, Tub-GAL80; 24F06-GAL4, UAS-mCD8GFP /+* |
| 6D | *hsFLP^122^/yw; FRT40, Tor*^Δ^*^P^/ FRT40, Tub-GAL80; DIP-*γ*-GAL4, UAS-mCD8GFP/UAS-Babo^DN^* |
| 6E | *hsFLP1, Tub-Gal80, FRT19A/FRT19A; +/+; Ort^C2b^-GAL4, UAS- myr-TdTomato/+* |
| 6F | *hsFLP1, Tub-Gal80, FRT19A/armLacZ, FRT19A; UAS-InR^wt^/UAS-mCD8GFP; DIP-*γ*-GAL4/+* |
| 6G | *hsFLP^122^, sev^E2^; FRT40, Pten^2L117^/ FRT40, Tub-GAL80; 24F06-GAL4, UAS-mCD8GFP/+* |
| 6H | *hsFLP^122^/yw; FRT40, Pten^2L117^/ FRT40, Tub-GAL80; DIP-*γ*-GAL4, UAS-mCD8GFP/UAS-Babo^DN^* |
| 6I | *hsFLP^122^/yw; FRT40, Pten^2L117^/ FRT40, Tub-GAL80; DIP-*γ*-GAL4, UAS-mCD8GFP/UAS-Babo^DA^* |
| 6J | *hsFLP^122^, sevE2; Ort^C2b^-GAL4, UAS-mCD8GFP/+; FRT82, Tsc1^1A2^/ FRT82, Tub-GAL80* |
| S1A-A” | *yw; UAS-mCD8GFP /+; 24F06-GAL4/+* |
| S1B-B’ | *hsFLP^122^/yw; FRT40/ FRT40, Tub-GAL80; 24F06-GAL4, UAS-mCD8GFP/+* |
| S1C-C’ | *hsFLP^122^/yw; FRT40, Tor*^Δ^*^P^/ FRT40, Tub-GAL80; 24F06-GAL4, UAS-mCD8GFP/+* |
| S1D-D’ | *hsFLP^122^/yw; FRT40, Pten^2L117^/ FRT40, Tub-GAL80; 24F06-GAL4, UAS-mCD8GFP/+* |
| S1F&H&J&L | *hsFLP^122^/yw; FRT40/ FRT40, Tub-GAL80; 24F06-GAL4, UAS-mCD8Cherry/+* |
| S1G&I&K&M | *hsFLP^122^/yw; FRT40, Tor*^Δ^*^P^/ FRT40, Tub-GAL80; 24F06-GAL4, UAS-mCD8Cherry/+* |
| S2A | *hsFLP^122^/yw; FRT40/ FRT40, Tub-GAL80; 24F06-GAL4, UAS-mCD8Cherry/+* |
| S2B | *hsFLP^122^/yw; FRT40, dock^k13421^/ FRT40, Tub-GAL80; 24F06-GAL4, UAS-mCD8Cherry/+* |
| S2C | *hsFLP^122^/yw; Ort^C2b^-GAL4, UAS-mCD8GFP/+; FRT82, foxo*^Δ^*^94^/ FRT82, Tub-GAL80* |
| S2D | *FRT19A, hsFLP^1^, Tub-GAL80/FRT19A, rictor*^Δ2^*; +; 24F06-GAL4, UAS-mCD8Cherry/+* |
| S2E | *hsFLP^122^/yw; Ort^C2b^-GAL4, UAS-mCD8-GFP/ +; FRT2A, S6K^l-1^/FRT2A, Tub-GAL80* |
| S2F | *hsFLP^122^/yw; Ort^C2b^-GAL4, UAS-mCD8GFP/+; FRT82, Thor*^k07736^*/ FRT82, Tub-GAL80* |
| S2G | *hsFLP^122^/yw; FRT40, Dref^KG09294^/ FRT40, Tub-GAL80; 24F06-GAL4, UAS-mCD8GFP/+* |
| S2H | *hsFLP^122^/yw; FRT40/ FRT40, Tub-GAL80; DIP-*γ*-GAL4, UAS-mCD8GFP/UAS-Tor^WT^* |
| S3B | *OrtC1a-GAL4/+; UAS-Ort-HA-spGFP11/UAS-mCD8mCherry* |
| S3C | *Rh3-Syb-spGFP1-10, Rh4-Syb-spGFP1-10/+* |
| S3D-D”’ | *hsFLP^122^/yw; FRT40/ FRT40, Tub-GAL80; 24F06-GAL4, UAS-mCD8Cherry /Rh3/4-Syb-spGFP1-10, UAS-Ort-HA-spGFP11* |
| S3E-E”’ | *hsFLP^122^/yw; FRT40, Tor*^Δ^*^P^/ FRT40, Tub-GAL80; 24F06-GAL4, UAS-mCD8Cherry /Rh3/4-Syb-spGFP1-10, UAS-Ort-HA-spGFP11* |
| S3F-F”’ | *hsFLP^122^/yw; FRT40, Pten^2L117^/ FRT40, Tub-GAL80; 24F06-GAL4, UAS-mCD8Cherry /Rh3/4-Syb-spGFP1-10, UAS-Ort-HA-spGFP11* |
| S3G-G” | *hsFLP^122^/yw; FRT40, chico^fs(2)4^/ FRT40,Tub-GAL80; 24F06-GAL4, UAS-mCD8Cherry /Rh3/4-Syb-spGFP1-10, UAS-Ort-HA-spGFP11* |
| S3H-H”” | *hsFLP^122^/yw; FRT40/ FRT40, Tub-GAL80; 24F06-GAL4, UAS-mCD8GFP /Rh4-Brp-Cherry* |
| S3I-I”” | *hsFLP^122^/yw; FRT40, Tor*^Δ^*^P^ / FRT40, Tub-GAL80; 24F06-GAL4, UAS-mCD8GFP /Rh4-Brp-Cherry* |
| S3J-J”” | *hsFLP^122^/yw; FRT40, Pten^2L117^/ FRT40, Tub-GAL80; 24F06-GAL4, UAS-mCD8GFP /Rh4-Brp-Cherry* |
| S4A-C’ | *GMR-GAL4, UAS-RFP; UAS-TdTomato-T2A-spGFP1-10 /+; InR-V5-spGFP11/+* |
| S5A-A”’ | *UAS-Dicer2/yw; +; Rh3/4-Syb-spGFP1-10/+* |
| S5B-B”’ | *UAS-Dicer2/yw; UAS-Dilp2-Ri/+; Rh3/4-Syb-spGFP1-10/6-60-GAL4* |
| S5C-C”’ | *UAS-Dicer2/yw; UAS-Dilp2-Ri/+; Rh3/4-Syb-spGFP1-10/GMR9B08-GAL4* |
| S5E-E” | *UAS-Dicer2/yw; UAS-Dilp2-Ri/+; Rh3/4-Syb-spGFP1-10/GMR9B08-GAL4* |
| S5F-F’ | *yw; UAS-mCD8GFP /+; Imp-L2-RA-GAL4/+* |
