# Supplementary material for: Antagonistic regulation by insulin-like peptide and activin ensures the elaboration of appropriate dendritic field sizes of amacrine neurons
Source: eLife. 2020 Mar 16;9:e50568. doi: 10.7554/eLife.50568 (PMC7075694; doi:10.7554/eLife.50568)
Supplement: Supplementary file 2. [file elife-50568-supp2.docx]

| Comparison | N1 | N2 | p value t-Test | Figure |
| --- | --- | --- | --- | --- |
| *wt And TorΔP* | 30 | 21 | 5.15E-16 | 1b |
| *wt And Tork17004* | 30 | 12 | 4.84E-09 | 1b |
| *wt And TorΔP rescue* | 30 | 21 | 0.045323651 | 1b |
| *wt And Rheb3M2* | 30 | 14 | 1.47E-07 | 1b |
| *wt And Tsc1Q87X* | 30 | 10 | 2.30E-15 | 1b |
| *wt And Tsc11A2* | 30 | 11 | 2.46E-08 | 1b |
| *TorΔP And TorΔP rescue* | 21 | 21 | 4.60E-15 | 1b |
| *Tork17004 And TorΔP rescue* | 12 | 21 | 3.13E-09 | 1b |
| *wt And InR273* | 30 | 15 | 6.93E-10 | 2n |
| *wt And InR353* | 30 | 16 | 2.42E-09 | 2n |
| *wt And chico1* | 30 | 14 | 1.18E-10 | 2n |
| *wt And chicofs(2)4* | 30 | 16 | 7.86E-20 | 2n |
| *wt And Pi3k92EA* | 30 | 12 | 1.84E-10 | 2n |
| *wt And raptorDel* | 30 | 30 | 1.16E-13 | 2n |
| *wt And Tor/ U-InRDN* | 30 | 27 | 1.61E-24 | 2n |
| *wt And U-InRDN* | 30 | 13 | 2.32E-19 | 2n |
| *wt And Pten2L117* | 30 | 24 | 3.93E-22 | 2n |
| *wt And SREBP189* | 30 | 16 | 2.60E-10 | 2n |
| *wt And U-SREBPWT* | 30 | 14 | 0.251861959 | 2n |
| *wt And U-SREBPCA* | 30 | 8 | 1.18E-11 | 2n |
| *TorΔP/U-InRDN And U-InRDN* | 27 | 13 | 0.954368181 | 2n |
| *TorΔP And U-SREBPWT* | 21 | 14 | 6.03E-09 | 2n |
| *TorΔP And TorΔP/ SREBPCA* | 21 | 4 | 0.822328127 | 2n |
| *wt And TorΔP/ SREBPWT* | 30 | 14 | 0.075139917 | 2n |
| *ctrl And LN9B08>Dilp2-Ri* | 18 | 16 | 1.99E-07 | 5p |
| *ctrl And LN27G05>Dilp2-Ri* | 18 | 15 | 0.007751386 | 5p |
| *ctrl And L56-60>Dilp2-Ri* | 18 | 20 | 9.04E-06 | 5p |
| *ctrl And GMR>Dilp2-Ri* | 18 | 14 | 0.974628159 | 5p |
| *ctrl And GMR>Dilp6-Ri* | 18 | 9 | 0.807207116 | 5p |
| *ctrl And L56-60>Dilp2* | 18 | 16 | 0.008597704 | 5p |
| *ctrl And GMR>Dilp2* | 18 | 12 | 0.328009552 | 5p |
| *ctrl And GMR>Dilp6* | 18 | 14 | 0.197497838 | 5p |
| *wt And sev/Tor* | 30 | 25 | 0.356659448 | 6i |
| *wt And BaboDN/Tor* | 30 | 28 | 0.995530979 | 6i |
| *wt And sev* | 30 | 18 | 2.46E-11 | 6i |
| *BaboDN/Tor And BaboDN* | 28 | 18 | 0.000228262 | 6i |
| *Pten And sev/Pten* | 24 | 8 | 0.040737138 | 6i |
| *Pten And BaboDN/Pten* | 24 | 34 | 0.271454269 | 6i |
| *Pten And baboDA/PTen* | 24 | 12 | 0.042695717 | 6i |
| *Tsc1 And sev/Tsc1* | 11 | 10 | 0.280267476 | 6i |
| *wt And Pten* | 39 | 21 | 1.35E-15 | S1e |
| *wt And Tor* | 39 | 20 | 0.045970105 | S1e |
| *wt And dockk13421* | 30 | 11 | 0.467774067 | S2j |
| *wt And foxoΔ94* | 30 | 15 | 0.233907363 | S2j |
| *wt And rictorΔ2* | 30 | 14 | 0.592053575 | S2j |
| *wt And s6kl-1* | 30 | 16 | 0.905082641 | S2j |
| *wt And Thork07736* | 30 | 11 | 0.583246124 | S2j |
| *wt And DrefKG09294* | 30 | 12 | 0.925015385 | S2j |
| *wt And U-TorWT* | 30 | 13 | 0.613293554 | S2j |
| *wt And Atg7d06996* | 30 | 8 | 0.454225404 | S2j |

| Comparison | N1 | N2 | p value Conover Test | p value Siegel-Tukey Test | Figure |
| --- | --- | --- | --- | --- | --- |
| *wt And Tor* | 30 | 21 | 0.587294724 | 0.759434404 | 6k |
| *wt And sev/Tor* | 30 | 25 | 0.000425180 | 0.004515339 | 6k |
| *wt And BaboDN/Tor* | 30 | 28 | 0.003398129 | 0.034324830 | 6k |
| *wt And BaboDN* | 30 | 18 | 0.004496667 | 0.004322030 | 6k |
| *wt And sev* | 30 | 18 | 0.002326241 | 0.000518081 | 6k |
| *wt And Pten* | 30 | 24 | 0.006227299 | 0.049174275 | 6k |
| *wt And Tsc1* | 30 | 11 | 0.004459023 | 0.015830133 | 6k |
| *wt And U-InR(wt)* | 30 | 21 | 2.02E-06 | 3.27919E-05 | 6k |
