# Supplementary material for: Antagonistic regulation by insulin-like peptide and activin ensures the elaboration of appropriate dendritic field sizes of amacrine neurons
Source: eLife. 2020 Mar 16;9:e50568. doi: 10.7554/eLife.50568 (PMC7075694; doi:10.7554/eLife.50568)
Supplement: Supplementary file 3. [file elife-50568-supp3.docx]

| **Key Resources Table** | | | | |
| --- | --- | --- | --- | --- |
| **Reagent type (species) or resource** | **Designation** | **Source or reference** | **Identifiers** | **Additional information** |
| antibody | Rat anti-RFP (monoclonal) | Bulldog Bio | ITEM# RMA5F8 | (1:500) |
| antibody | Mouse Ig1 anti-24B10 (monoclonal) | Developmental Studies Hybridoma Bank (DSHB) | ID# AB_528161 | (1:20) |
| antibody | Mouse Ig1 anti-GFP-G1 (monoclonal) | DSHB | ID# AB_ 2619561 | (1:500) |
| antibody | Mouse Ig2b anti-Highwire (monoclonal) | DSHB | ID# AB_528277 | (1:20) |
| antibody | Mouse Ig2a anti-eyeless (monoclonal) | DSHB | ID# AB_2253542 | (1:20) |
| antibody | Mouse Ig2a anti-dachshund (monoclonal) | DSHB | ID# AB_579773 | (1:100) |
| antibody | Rat anti-Elav (monoclonal) | DSHB | ID# [AB_528218](http://antibodyregistry.org/AB_528218) | (1:100) |
| antibody | Guinea pig anti-Dichaete | Gift from Claude Desplan |  | (1:50) |
| antibody | Guinea pig anti-Bsh | Gift from Makoto Sato |  | (1:600) |
| antibody | Rat anti-dilp2 | Gift from Pierre Leopold |  | (1:500) |
| antibody | Rat anti-drifter | Gift from Sarah J Certel |  | (1:1000) |
| antibody | Guinea pig anti-Toy | Gift from Uwe Walldorf |  | (1:100) |
| antibody | Rat anti-HA (monoclonal) | Sigma-Aldrich | Cat# 11867423001 | (1:500) |
| antibody | Rabbit anti-GFP (monoclonal) | Thermo Fisher Scientific | Cat# G-10362 | (1:500) |
| antibody | Rabbit anti-GFP (polyclonal) | Thermo Fisher Scientific | Cat# A-11122 | (1:2000) |
| antibody | Mouse Ig2a anti-V5 (monoclonal) | Thermo Fisher Scientific | Cat# R960-25 | (1:500) |
| antibody | Mouse Ig2a anti-GFP (monoclonal) | Thermo Fisher Scientific | Cat# A-11120 | (1:500) |
| antibody | Rat anti-CD8 (monoclonal) | Thermo Fisher Scientific | Cat# MCD0800 | (1:500) |
| antibody | Goat anti-rabbit Alexa 488 | Thermo Fisher Scientific | Cat# A-11034 | (1:500) |
| antibody | Goat anti-rabbit Alexa 568 | Thermo Fisher Scientific | Cat# A-11036 | (1:500) |
| antibody | Goat anti-rabbit Alexa 647 | Thermo Fisher Scientific | Cat# A-21245 | (1:500) |
| antibody | Goat anti-mouse Ig1 Alexa 488 | Thermo Fisher Scientific | Cat# A-21121 | (1:500) |
| antibody | Goat anti-mouse Ig1 Alexa 568 | Thermo Fisher Scientific | Cat# A-21124 | (1:500) |
| antibody | Goat anti-mouse Ig1 Alexa 647 | Thermo Fisher Scientific | Cat# A-21240 | (1:500) |
| antibody | Goat anti-mouse Ig2a Alexa 488 | Thermo Fisher Scientific | Cat# A-21131 | (1:500) |
| antibody | Goat anti-mouse Ig2a Alexa 568 | Thermo Fisher Scientific | Cat# A-21134 | (1:500) |
| antibody | Goat anti-mouse Ig2a Alexa 647 | Thermo Fisher Scientific | Cat# A-21241 | (1:500) |
| antibody | Goat anti-mouse Ig2b Alexa 488 | Thermo Fisher Scientific | Cat# A-21141 | (1:500) |
| antibody | Goat anti-mouse Ig2b Alexa 568 | Thermo Fisher Scientific | Cat# A-21144 | (1:500) |
| antibody | Goat anti-mouse Ig2b Alexa 647 | Thermo Fisher Scientific | Cat# A-21242 | (1:500) |
| antibody | Goat anti-guinea pig Alexa 488 | Thermo Fisher Scientific | Cat# A-11073 | (1:500) |
| antibody | Goat anti-guinea pig Alexa 568 | Thermo Fisher Scientific | Cat# A-11075 | (1:500) |
| antibody | Goat anti-guinea pig Alexa 647 | Thermo Fisher Scientific | Cat# A-21450 | (1:500) |
| antibody | Goat anti-rat Alexa 568 | Thermo Fisher Scientific | Cat# A-11077 | (1:500) |
| antibody | Goat anti-rat Alexa 647 | Thermo Fisher Scientific | Cat# A-21247 | (1:500) |
| chemical compound, drug | PCR Master Mix | Thermo Fisher Scientific | Cat# K0171 |  |
| chemical compound, drug | Low-melting point agarose | Thermo Fisher Scientific | Cat# 16520050 |  |
| chemical compound, drug | Protease K | Thermo Fisher Scientific | Cat#EO0491 |  |
| chemical compound, drug | G418 | Sigma Aldrich | Cat# G5013 |  |
| chemical compound, drug | GreenGlo DNA dye | Denville Scientific | Cat# CA3600 |  |
| chemical compound, drug | VECTASHIELD | Vector Laboratories | Cat# H1000; RRID: AB_2336789 |  |
| chemical compound, drug | Paraformaldehyde 20% Solution | Electron Microscopy Sciences | Cat# 15713-S |  |
| chemical compound, drug | Restriction enzymes | New England Biolabs |  |  |
| chemical compound, drug | DNA Gel Loading Solution (5x) | Quality Biological | Cat# 351-028-661 |  |
| chemical compound, drug | TAE (10x) | Crystalgen | Cat# 221-197-01 |  |
| commercial assay or kit | Nucleobond Midiprep Kit | Promega | Cat# 740420.10 |  |
| Biological sample | Xenopus oocyte | EcoCyte |  |  |
| genetic reagent (*D. melanogaster*) | hsFLP1 | Bloomington *Drosophila* Stock Center (BDSC) | BDSC_6 |  |
| genetic reagent (*D. melanogaster*) | hsFLP122 | BDSC | BDSC_23649 |  |
| genetic reagent (*D. melanogaster*) | GMR-Gal4 | BDSC | BDSC_1104 |  |
| genetic reagent (*D. melanogaster*) | longGMR-Gal4 | BDSC | BDSC_8121 |  |
| genetic reagent (*D. melanogaster*) | Actin5c-Gal4 | BDSC | BDSC_4414 |  |
| genetic reagent (*D. melanogaster*) | GMR24F06-Gal4 | BDSC | BDSC_49087 |  |
| genetic reagent (*D. melanogaster*) | GMR27G05-Gal4 | BDSC | BDSC_48073 |  |
| genetic reagent (*D. melanogaster*) | GMR9B08-Gal4 | BDSC | BDSC_41369 |  |
| genetic reagent (*D. melanogaster*) | GMR24F06-LexA | BDSC | BDSC_52695 |  |
| genetic reagent (*D. melanogaster*) | UAS-mCD8::GFP | BDSC | BDSC_32184 |  |
| genetic reagent (*D. melanogaster*) | UAS-mCD8::mCherry | BDSC | BDSC_27392 |  |
| genetic reagent (*D. melanogaster*) | UAS-Tor^wt^ | BDSC | BDSC_7012 |  |
| genetic reagent (*D. melanogaster*) | UAS-InR^wt^ | BDSC | BDSC_8262 |  |
| genetic reagent (*D. melanogaster*) | UAS-InR^K1409A^ | BDSC | BDSC_8253 |  |
| genetic reagent (*D. melanogaster*) | InR deletion Df(3R)ED6058 | BDSC | BDSC_24140 |  |
| genetic reagent (*D. melanogaster*) | In(3R)GC25, InR^93Dj-4^ | BDSC | BDSC_9554 |  |
| genetic reagent (*D. melanogaster*) | FRT2A, SREBP^189^ | BDSC | BDSC_38392 (Kunte et al., 2006) |  |
| genetic reagent (*D. melanogaster*) | FRT40,Thor^k07736^ | Kyoto Stock Center | 114605  (Spradling et al., 1999) |  |
| genetic reagent (*D. melanogaster*) | FRT40, Atg7^d06996^ | Kyoto Stock Center | 114560 |  |
| genetic reagent (*D. melanogaster*) | FRT40,Dref^KG09294^ | Kyoto Stock Center | 114408  (Bellen et al., 2004) |  |
| genetic reagent (*D. melanogaster*) | FRT82, S6k^l-1^ | BDSC | BDSC_32552  (Montagne et al., 1999) |  |
| genetic reagent (*D. melanogaster*) | FRT82, Df(3R)PI3K92E^A^ | BDSC | BDSC_25900 (Weinkove et al.,1999) |  |
| genetic reagent (*D. melanogaster*) | Imp-L2-RA-Gal4 | Gift from Ernst Hafen | (Bader et al., 2013) |  |
| genetic reagent (*D. melanogaster*) | UAS-Dilp2 | Gift from Ernst Hafen | (Ikeya et al., 2002) |  |
| genetic reagent (*D. melanogaster*) | UAS-Dilp6 | Gift from Ernst Hafen | (Ikeya et al., 2002) |  |
| genetic reagent (*D. melanogaster*) | FRT40,Chico^1^ | Gift from Ernst Hafen | (Bohni et al., 1999) |  |
| genetic reagent (*D. melanogaster*) | FRT40,Chico^fs(2)4^ | Gift from Ernst Hafen | (Bohni et al., 1999) |  |
| genetic reagent (*D. melanogaster*) | FRT40,Pten^2L117^ | Gift from Ernst Hafen | (Oldham et al.,2002) |  |
| genetic reagent (*D. melanogaster*) | FRT82,Dp110^1C1^ | Gift from Ernst Hafen | (Willecke et al., 2011) |  |
| genetic reagent (*D. melanogaster*) | FRT82,Rheb^3M2^ | Gift from Ernst Hafen | (Stocker et al.,2003) |  |
| genetic reagent (*D. melanogaster*) | FRT82,Tsc1^1A2^ | Gift from Ernst Hafen | (Stocker et al.,2003) |  |
| genetic reagent (*D. melanogaster*) | Dilp2-Gal4 | Gift from Edwin Levitan | (Rulifson et al.,2002) |  |
| genetic reagent (*D. melanogaster*) | UAS-Dilp2::GFP | Gift from Edwin Levitan | (Wong et al., 2012) |  |
| genetic reagent (*D. melanogaster*) | UAS-IVS-R::PEST | Gift from Gerrald Rubin | (Nern et al, 2011) |  |
| genetic reagent (*D. melanogaster*) | FRT19A, rictor^Δ2^ | Gift from Ville Hietakangas | (Hietakangas et al, 2007) |  |
| genetic reagent (*D. melanogaster*) | FRT19A, Raptor^Del^ | Gift from Jianzhong Yu | (Li et al., 2019) |  |
| genetic reagent (*D. melanogaster*) | DIP-γ-Gal4 | Gift from Kai Zinn | (Carrillo et al, 2015) |  |
| genetic reagent (*D. melanogaster*) | 6-60-Gal4 | Gift from Larry Zipursky | (Nern et al., 2008) |  |
| genetic reagent (*D. melanogaster*) | FRT40,Tor^ΔP^ | Gift from Mary Lily | (Zhang et al., 2000) |  |
| genetic reagent (*D. melanogaster*) | FRT82,Tsc1^Q87X^ | Gift from Mary Lily | (Tapon 2001 Cell) |  |
| genetic reagent (*D. melanogaster*) | FRT82, InR273 | Gift from Leslie Pick | (Song et al., 2003) |  |
| genetic reagent (*D. melanogaster*) | FRT82,InR^353^ | Gift from Leslie Pick | (Song et al., 2003) |  |
| genetic reagent (*D. melanogaster*) | InR^E19^ | Gift from Hwei-Jan Hsu | (Kao et al., 2015) |  |
| genetic reagent (*D. melanogaster*) | InR^339^ | Gift from Hwei-Jan Hsu | (Kao et al., 2015) |  |
| genetic reagent (*D. melanogaster*) | arm-lacZ, FRT19A | Gift from Hwei-Jan Hsu |  |  |
| genetic reagent (*D. melanogaster*) | FRT82,Foxo^Δ94^ | Gift from Linda Partridge | (Slack et al., 2011) |  |
| genetic reagent (*D. melanogaster*) | Rh4-Brp::short^mCherry^ | Gift from Takashi Suzuki | (Berger-Muller et al., 2013) |  |
| genetic reagent (*D. melanogaster*) | UAS-CD4::spGFP1-10 | Gift from Kristin Scott | (Gordon et al., 2009) |  |
| genetic reagent (*D. melanogaster*) | GMR-CD4::spGFP11 | Lee Lab | (Ting et al., 2014) |  |
| genetic reagent (*D. melanogaster*) | Ort^C2b^-Gal4 | Lee Lab | (Gao et al., 2008) |  |
| genetic reagent (*D. melanogaster*) | Ort^C1a^-Gal4 | Lee Lab | (Karuppudurai et al., 2014) |  |
| genetic reagent (*D. melanogaster*) | Dm8-LexA | Lee Lab | (Karuppudurai et al., 2014) |  |
| genetic reagent (*D. melanogaster*) | Rh3-Syb::spGFP1-10 | This study |  | Materials & Methods Section |
| genetic reagent (*D. melanogaster*) | Rh4-Syb::spGFP1-10 | This study |  | Materials & Methods Section |
| genetic reagent (*D. melanogaster*) | UAS-Ort::HA::spGFP11 | This study |  | Materials & Methods Section |
| genetic reagent (*D. melanogaster*) | UAS-FSF-tdTom::T2A::spGFP1-10 | This study |  | Materials & Methods Section |
| genetic reagent (*D. melanogaster*) | InR::V5::spGFP11 | This study |  | Materials & Methods Section |
| genetic reagent (*D. melanogaster*) | UAS-Dilp2-RNAi | Vienna *Drosophila* Research Center (VDRC) | VDRC_102158 |  |
| genetic reagent (*D. melanogaster*) | UAS-Dilp6-RNAi | VDRC | VDRC_ 102465 |  |
| genetic reagent (*D. melanogaster*) | UAS-Babo^DN^ | Gift from Tzu-Min Lee |  |  |
| genetic reagent (*D. melanogaster*) | UAS-Babo^DA^ | BDSC | BDSC_64293 |  |
| genetic reagent (*D. melanogaster*) | UAS-SREBP^WT^ | BDSC | BDSC_8236 |  |
| genetic reagent (*D. melanogaster*) | UAS-SREBP^CA^ | BDSC | BDSC_8242 |  |
| genetic reagent (*D. melanogaster*) | LexAop-mCD8::GFP | Gift from Gerrald Rubin | (Pfeiffer et al., 2010) |  |
| genetic reagent (*D. melanogaster*) | LexAop-FSF-mCD8::GFP | Lee lab | (Ting et al., 2014) |  |
| genetic reagent (*D. melanogaster*) | LexAop-FSF-rCD2::mCherry | Lee lab | (Ting et al., 2014) |  |
| genetic reagent (*D. melanogaster*) | *Sev^E2^* | Lee lab | (Ting et al., 2014) |  |
| sequence-based reagent | InR tagging | This paper (custom made by Operon) | PCR primers | For 5’ homologous fragment of InR:  Fwd:  5’-CGAATGCATCTAGATATCGACTTACGTTGTGC  CTGATGC-3’  Rev:  5’-GGGATAGGAGCCGATATCCGCCTCCCTTCCG  ATGAATC-3’  For 3’ homologous fragment of InR:  Fwd1:  5’-TTCTAGGGTTAATACGTATCGTTACGAACTGT  AGTTCTGTAG-3’  Rev1:  5’-GTGTCATGGATCTACGGAAAGGTGTGTTATTT  TACGAAG-3’  Fwd2:  5’-CGTAGATCCATGACACCTTTTCTTCTTGGTGT  CGAACCA-3’  Rev2:  5’-CCGGGATCCGATTACGTAGAAAGTGGTATTC  CTAGAGCAGAGC-3’ |
| sequence-based reagent | For pCaST-Rh3::Syb::spGFP1-10: | This paper (custom made by Operon) |  | Fwd:  5’-AGGGCGAATTCGTTTAAACATGGCGGACGCT  GCAC-3’  Rev:  5’-ACAAAGATCCTCTAGACTATGTTCCTTTTTCAT  TTGGATCTTTGCT-3’  For pCaST-Rh4::Syb::spGFP1-10:  Fwd:  5’-CGGGTTGACCGGTTTAAACATGGCGGACGCT  GCAC-3’  Rev:  5’-ACAAAGATCCTCTAGACTATGTTCCTTTTTCAT  TTGGATCTTTGCT-3’ |
| sequence-based reagent | for UAS-spGFP11::HA::Ort | This paper (custom made by Operon) |  | 5’-TTCAACAAAGTCTGGCCATACGCGACCACAT  GGTGCTGCACGAGTACGTGAACGCCGCCGGCATCACCGGCTCCTACCCCTACGACGTGCCCGACTACGCCGGCTATCCCTATGACGTCCCGGACTATGCAGGCTCCTTGGCCATAACCGACATCC-3’ |
| sequence-based reagent | For UAS-FSF-myr::TdTom::dT2A::R::PEST | This paper (custom made by Operon) |  | Fwd:  5’-GGCCCTGGTAGCAGGCCTATGCAGCTTACCA  AGGACAC-3’  Rev:  5’-AATTCGTTTAAACGGCCGGCCAGATCGATCC  AGACATGATAAGATAC-3’ |
| sequence-based reagent | Probe synthesis for *in situ* hybridization | This paper (custom made by Operon) |  | T3-dilp2-sense Forward:  AATTAACCCTCACTAAAGGGCGGCTCGACCCAACTTAATC  dilp2-sense Reverse:  CGCGCTTGTGTGGAATCAC  dilp2-a-sense Forward:  CGGCTCGACCCAACTTAATC  T7-dilp2-antisense Reverse: TAATACGACTCACTATAGGGCGCGCTTGTGTGGAATCAC  T3-dilp2-sense-2 Forward: AATTAACCCTCACTAAAGGGGATCTGGACGCCCTCAATCC  dilp2-sense-2 Reverse:  CAGAGATAATCGCGTCGACC  dilp2-antisense-2 Forward:  GATCTGGACGCCCTCAATCC  T7-dilp2-antisense-2 Reverse: TAATACGACTCACTATAGGGCAGAGATAATCGCGTCGACC |
| recombinant DNA reagent | pUC57-InR::V5::spGFP11 | This paper |  | Maintained in Lee lab |
| recombinant DNA reagent | pCaST-Rh3(Rh4)::Syb::spGFP1-10 | This paper |  | Maintained in Lee lab |
| recombinant DNA reagent | pCaST-Rh3(Rh4)::Syb::spGFP1-10 | This paper |  | Maintained in Lee lab |
| recombinant DNA reagent | pUAST-spGFP11::HA::Ort | This paper |  | Maintained in Lee lab |
| recombinant DNA reagent | pUAST-myr::TdTom::dT2A::spGFP1-10 | This paper |  | Maintained in Lee lab |
| recombinant DNA reagent | pUAST-FSF-myr::TdTom::dT2A::spGFP1-10 | This paper |  | Maintained in Lee lab |
| recombinant DNA reagent | pUAST-FSF-myr::TdTom::dT2A::R::PEST | This paper |  | Maintained in Lee lab |
| software | Fiji/Image J | NIH | https://imagej.net/Fiji;RRID:SCR_002285 |  |
| software | Imaris 8.0 | Bitplane | http://www.bitplane.com/ |  |
| software | GraphPad Prism 7.0 | GraphPad Software | https://www.graphpad.com/; RRID: SCR_002798 |  |
| software | HuygensDeconvolution | Scientific Volume Imaging | https://svi.nl/ |  |
